# Supplementary material for: The ERAD Pathway Participates in Fungal Growth and Cellulase Secretion in Trichoderma reesei
Source: J Fungi (Basel). 2023 Jan 4;9(1):74. doi: 10.3390/jof9010074 (PMC9862206; doi:10.3390/jof9010074)
Supplement: Supplementary file 1 [file jof-09-00074-s001.zip › jof-2092949-supplementary.pdf]

Table S1 Primers used in this study.

Primers	Nucleotide sequence（5’ to 3’）	Employment
hrd1-UF	CTGCGTGTCTCAGTGGTAGGT	*hrd1* deletion strain construction
hrd1-UR	GGAGATGTTGCTGAAGTCGCCATTTCACGCAATCAGGTTC	*hrd1* deletion strain construction
hrd1-DF	ATGAGTCGTTTACCCAGAATCATGGCGACGTTTAATAGCA	*hrd1* deletion strain construction
hrd1-DR	GAAGTCTTCCGCACTGTTGAT	*hrd1* deletion strain construction
hrd3-UF	CCTGTGAGCTTCAGACCCAGTA	*hrd3* deletion strain construction
hrd3-UR	GGAGATGTTGCTGAAGTCGTATGCTGAGCCTGCCTTCTGT	*hrd3* deletion strain construction
hrd3-DF	ATGAGTCGTTTACCCAGAATAGGTGCTTGAGGAGGTTTGTC	*hrd3* deletion strain construction
hrd3-DR	CTCTTCCATCGTAGTGCGTTTC	*hrd3* deletion strain construction
der1-UF	CAGCACGGTAGACTGCCAGAT	*der1* deletion strain construction
der1-UR	GGAGATGTTGCTGAAGTCGAATGATGGAATGAATGCCTCTG	*der1* deletion strain construction
der1-DF	ATGAGTCGTTTACCCAGAATTTGTGCTGGAATACCTACTGCC	*der1* deletion strain construction
der1-DR	GATCCGGGACCTCGTTGAATA	*der1* deletion strain construction
hph-F	CGACTTCAGCAACATCTCC	*hph* markers amplifcation
hph-R	ATTCTGGGTAAACGACTCAT	*hph* markers amplifcation
hrd1-2991-UF	CGGCACAAGGTCACAGTTCAT	*hrd1* upstream anchoring validation primer
hrd1-5646-DR	GGCGACCCTACACGACCA	*hrd1* downstream anchoring validation primer
hrd1-F	CAGCAACCTCCTGCGAATC	*hrd1* gene validation primer
hrd1-R	TCCGCTTGCTGTAGAAGAGTT	*hrd1* gene validation primer
hrd3-2939-UF	CTCCGACTTCATTTGCCTGTG	*hrd3* upstream anchoring validation primer
hrd3- 4851-DR	TCGGCTCTTCGCTAACACG	*hrd3* downstream anchoring validation primer
hrd3-F	TTCTCAGGACCATCCCAACC	*hrd3* gene validation primer
hrd3-R	TGGCTATCCACTCTGAGAGGG	*hrd3* gene validation primer
der1-2926-UF	CTCGAAGGCTAGGGTCAGGT	*der1* upstream anchoring validation primer
der1-3501-DR	AATTTTCTCGCGGGATATGC	*der1* downstream anchoring validation primer
der1-F	GCCTCCCGCTGGAAACAT	*der1* gene validation primer
der1-R	CCTGCGGCCTTCAAACAA	*der1* gene validation primer
hph-934-UR	GTGGAGGCGGCGGATTTTA	Upstream anchoring validation primer
hph-608-DF	GAATGCTCCGTAACACCCAAT	Downstream anchoring validation primer
actin-F1	CCCAAGTCCAACCGTGAGA	Used for RT-PCR
actin-R1	CAATGGCGTGAGGAAGAGC	Used for RT-PCR
bip1-qF1	GATGCCAACGGTATCCTCA	Used for RT-PCR
bip1-qR1	TGCGGTCAATCTCCTCCT	Used for RT-PCR
pdi1-qF1	GTTGTCGTTGCCCACTCTTAC	Used for RT-PCR
pdi1-qR1	AGTCGCTCTTGGCATACAGG	Used for RT-PCR
hrd1-qF1	CCCAATGATGCCAAACTG	Used for RT-PCR
hrd1-qR1	TGCTGCTTTCAGGTGGAG	Used for RT-PCR
hrd3-qF1	AACGACGCATACGAGGCT	Used for RT-PCR
hrd3-qR1	CGCATCATCTCATAGAAGGGT	Used for RT-PCR
der1-qF1	CCTCGTTTACATTTGGTCTCG	Used for RT-PCR
der1-qR1	GCCCATGATCTCATCCCTC	Used for RT-PCR
cbh1-qF1	GCGGCATGGTTCTGGTCA	Used for RT-PCR
cbh1-qR1	TCGTTTGTCGGGTAGGTGGA	Used for RT-PCR
eg1-qF1	GGCTCGCTCTACCTGTCTCA	Used for RT-PCR
eg1-qR1	GGGTGCCGTTCCTCCAT	Used for RT-PCR
eg2-qF1	ACGAGCCTTTGGTCGCAGTT	Used for RT-PCR
eg2-qR1	GGCAGCCCAGGTGTTGATGT	Used for RT-PCR
bgl1-qF1	TCGATCCATATCTCACGGGC	Used for RT-PCR
bgl1-qR1	AGTTCGGTCATCTGGGTTGC	Used for RT-PCR

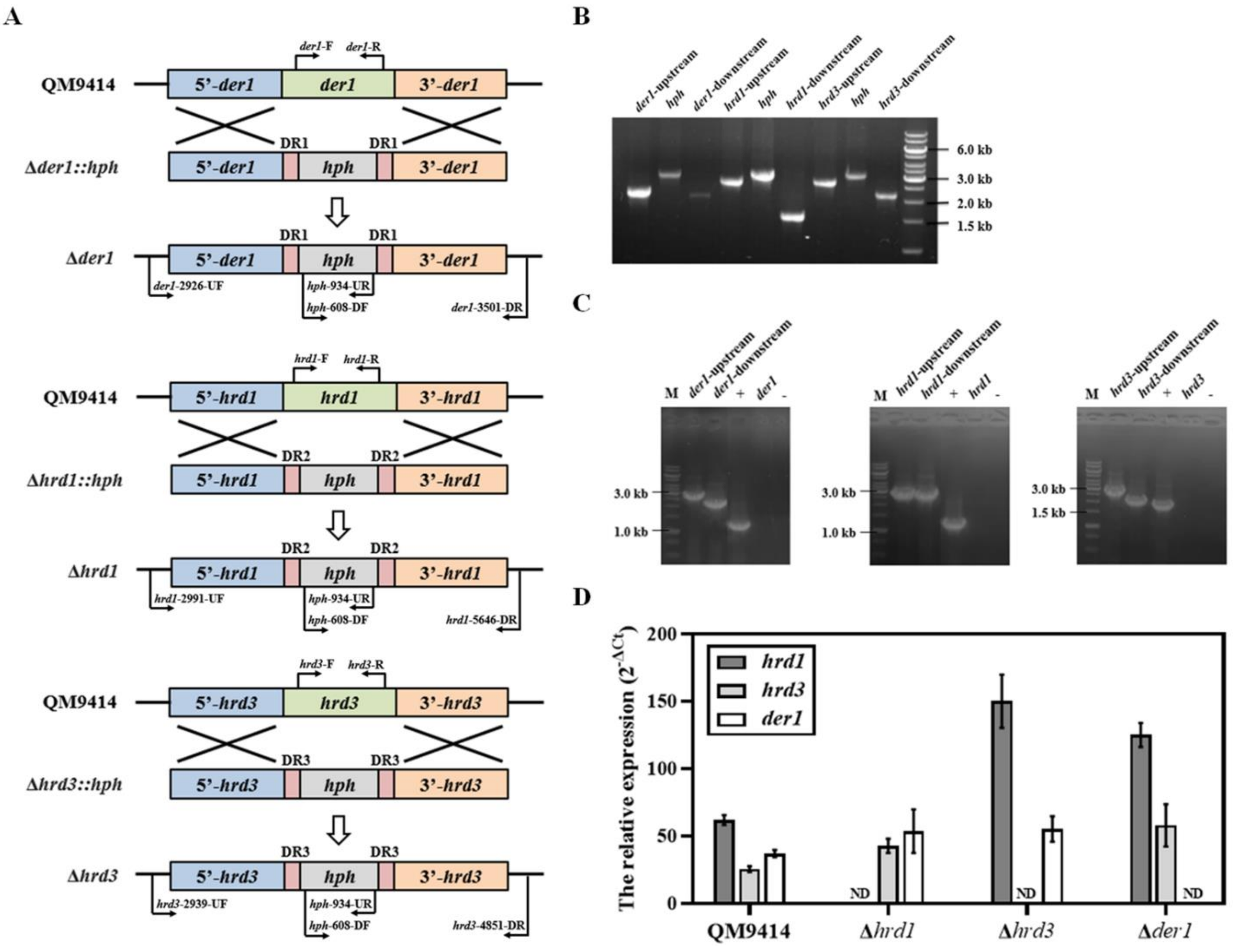

Fig. S1. Construction and verification of ERAD deletion strains Δ*der1*, Δ*hrd1* and Δ*hrd3*. (A) Sketch map of knocking out *der1*/*hrd1*/*hrd3* in *T. reesei* QM9414 was shown. The target gene was replaced by the resistance gene *hph* to obtain ERAD deletion strain through homologous recombination. The primers used to verify the upstream and downstream anchoring as well as knockout gene of the strains were shown. (B) PCR analysis showed that the *der1*/*hrd1*/*hrd3* knockout cassettes were successfully constructed. The *der1* knockout cassette contained 5' flanking sequence (2.3 kb), 3' flanking sequence (2.1 kb) and *hph* (2.8 kb). The *hrd1* knockout cassette contained 5' flanking sequence (2.3 kb), 3' flanking sequence (1.5 kb) and *hph* (2.8 kb). The *hrd3* knockout cassette contained 5' flanking sequence (2.5 kb), 3' flanking sequence (2.1 kb) and *hph* (2.8 kb). (C) The PCR analysis showed the knockout of the *der1*/*hrd1*/*hrd3* gene in the corresponding strains. The knockout strain contained the expected upstream and downstream anchoring, and did not contain the knockout gene. The *der1* knockout strain contained upstream anchoring (2.5 kb) and downstream anchoring (2.3 kb), but did not contain *der1* (1.1 kb). The *hrd1* knockout strain contained upstream anchoring (2.5 kb) and downstream anchoring (2.5 kb), but did not contain *hrd1* (1.1 kb). The *hrd3* knockout strain contained upstream anchoring (2.7 kb) and downstream anchoring (2.4 kb), but did not contain *hrd3* (1.9 kb). "+" represented the knockout gene amplified from the QM9414 genome and served as a positive control. "-" represented the knockout gene amplified without template, as a negative control. (D) The transcription levels of *der1*/*hrd1*/*hrd3* in the ERAD-deficient mutants Δ*der1*, Δ*hrd1* and Δ*hrd3*. The results showed that the transcripts of the knockout genes could not be detected in the corresponding strains. Values represent the mean of three repeated measurements taken from at least three parallel experiments. The error bars refer to the standard deviations. ND: not detected.

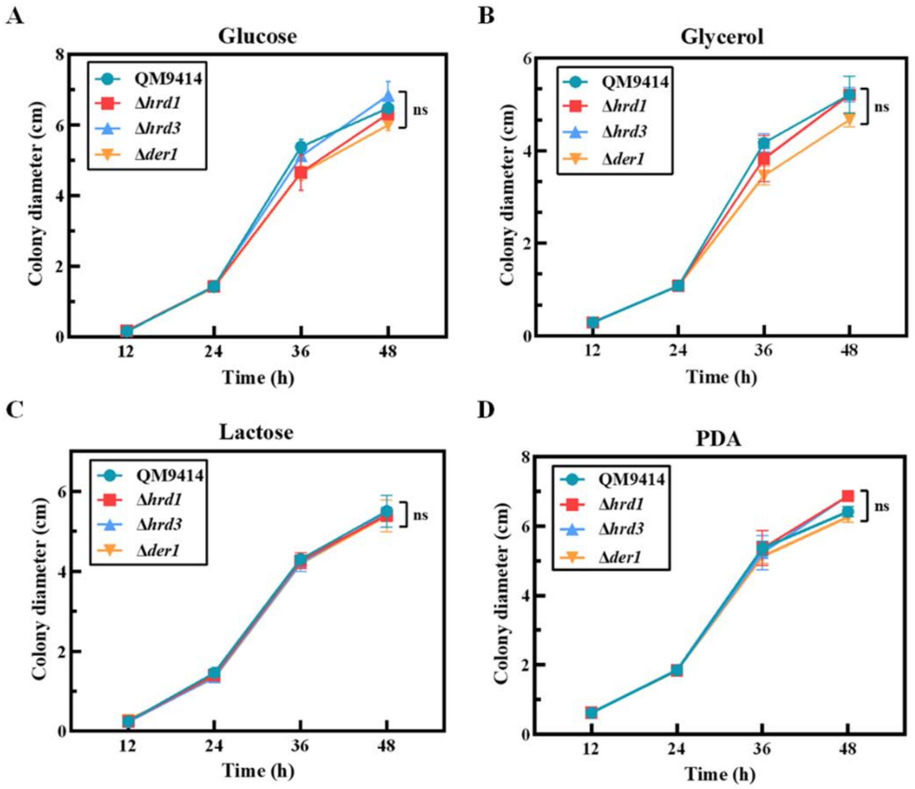

Fig. S2. Radial extension rates of the ERAD-deficient mutants on different carbon sources. Radial extension rates of mycelium were measured after cultivation on the following carbon sources: glucose (A), glycerol (B), lactose (C), and PDA (D) at 30°C for 2 days. The colony diameters were measured every 12h to draw the mycelial growth rate map. Values represent the mean of three repeated measurements taken from at least three parallel experiments. The error bars refer to the standard deviations. The difference between the parental strain and the knockout strains was shown by analysis of ANOVA followed by Turkey test. ns = not significant. Circles, QM9414; squares, Δ*hrd1*; upper triangle, Δ*hrd3*; lower triangles, Δ*der1*.

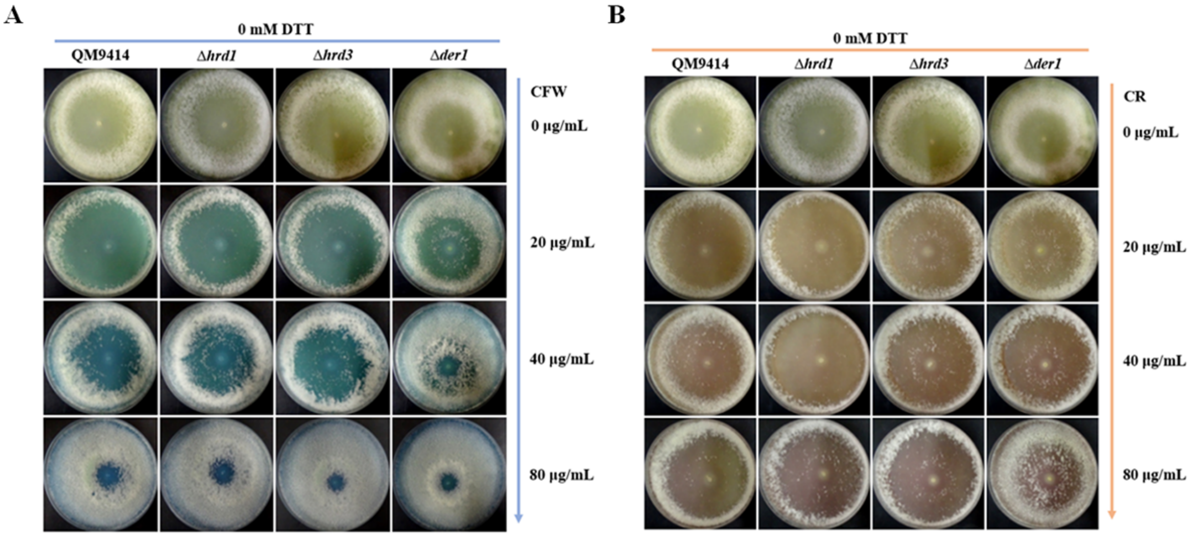

Fig. S3. Cell wall stability of the ERAD-deficient mutants without ER pressure. Growth analysis of QM9414 and the ERAD-deficient mutants using the MM plates supplemented with different concentrations of Calcofluor White (A) or Congo red (B). The concentrations of CFW or CR were 0 μg/mL, 20 μg/mL, 40 μg/mL, and 80 μg/mL, respectively. The strains were growth on MM containing glucose as carbon source at 30 ℃ for 3 days and its growth state was recorded by photographing.

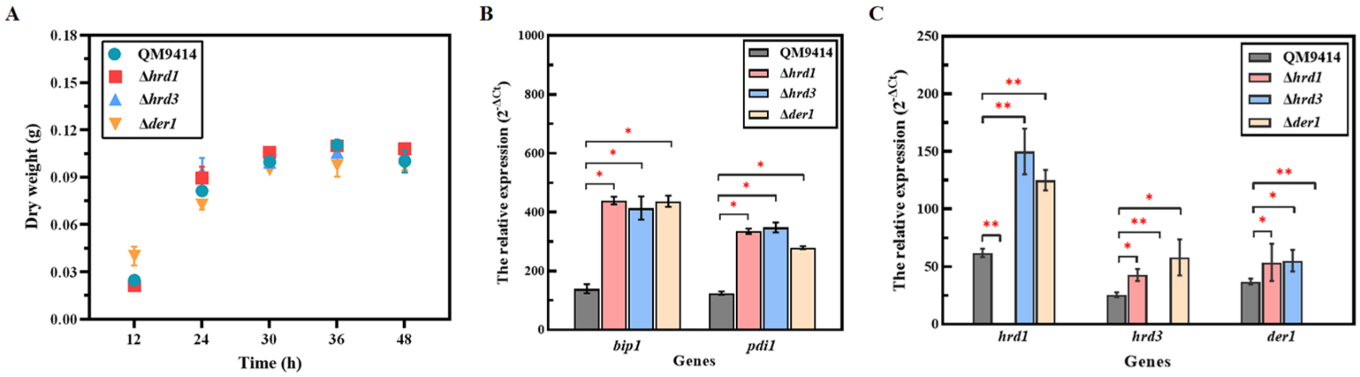

Fig. S4. Growth curve and ER pressure of the ERAD-deficient mutants under normal growth conditions. (A) The growth curve of QM9414 and ERAD-deficient mutants was made by measuring the dry weight of hyphae obtained after MM liquid culture to determine the logarithmic growth phase. All strains were cultured in MM liquid medium containing glucose as carbon source, and the dry weight was measured every 12 hours. (B) The relative transcription levels of UPR-related genes *bip1* and *pdi1* in the ERAD-deficient mutants under normal growth conditions. (C) The relative transcription levels of *hrd1*/*hrd3*/*der1* in the ERAD-deficient mutants under normal growth conditions. *Actin* gene were universally used to normalize gene transcription levels. Values represent the mean of three repeated measurements taken from at least three parallel experiments. The error bars refer to the standard deviations. The difference between the parental strain and the knockout strains was shown by analysis of ANOVA followed by Turkey test. *P < 0.05; **P < 0.01.
